# Investigation of Permeation of Theophylline through Skin Using Selected Piperazine-2,5-Diones

**DOI:** 10.3390/molecules24030566

**Published:** 2019-02-04

**Authors:** Aneta Pokorna, Pavel Bobal, Michal Oravec, Lucie Rarova, Janette Bobalova, Josef Jampilek

**Affiliations:** 1 Department of Chemical Drugs, Faculty of Pharmacy, University of Veterinary and Pharmaceutical Sciences Brno, Palackeho 1946/1, 612 42 Brno, Czech Republic; pokorna.anetaa@gmail.com; 2 Global Change Research Institute CAS, Belidla 986/4a, 603 00 Brno, Czech Republic; oravec.m@czechglobe.cz; 3 Department of Chemical Biology and Genetics, Centre of the Region Hana for Biotechnological and Agricultural Research, Faculty of Science, Palacky University, Slechtitelu 27, 783 71 Olomouc, Czech Republic; lucie.rarova@upol.cz; 4 Department of Neurology, University Hospital in Olomouc, I. P. Pavlova 6, 775 20 Olomouc, Czech Republic; 5 Institute of Analytical Chemistry of the CAS, Veveri 97, 602 00 Brno, Czech Republic; bobalova@iach.cz; 6 Regional Centre of Advanced Technologies and Materials, Palacky University, Slechtitelu 27, 783 71 Olomouc, Czech Republic

**Keywords:** piperazine-2,5-diones, theophylline, permeation, skin, Franz diffusion cell, HPLC determination, cytotoxicity

## Abstract

Transdermal administration of drugs that penetrate, in this case directly into the blood circulation, has many advantages and is promising for many drugs thanks to its easy application and good patient compliance. (*S*)-8-Methyl-6,9-diazaspiro[4.5]decan-7,10-dione (alaptide), has been studied as a potential chemical permeation enhancer. Based on its structure, four selected piperazine-2,5-diones were synthesized by means of multi-step synthetic pathways. All the compounds were investigated on their ability to enhance the permeation of the model drug theophylline from the hydrophilic medium propylene glycol:water (1:1). In vitro experiments were performed using vertical Franz diffusion cells at constant temperature 34 ± 0.5 °C and using full-thickness pig (*Sus scrofa* f. *domestica*) ear skin. Withdrawn samples were analyzed by RP-HPLC for determination of the permeated amount of theophylline. All the compounds were applied in ratio 1:10 (*w*/*w*) relative to the amount of theophylline. One hour after application, the permeated amount of theophylline from formulations with alaptide and (3*S*,6*S*)-3,6-dimethylpiperazine-2,5-dione, was ca. 15- and 12-fold higher, respectively, than from the formulation without the tested compounds. Despite the enhancement ratio of both enhancers in a steady state was ca. 2.3, the pseudo-enhancement ratio in the time range from 1 to 3 h was 4.4. These enhancement ratios indicate that the compounds are able to enhance the permeation of agents through the skin; however, the short-term application of both compound formulations seems to be more advantageous. In addition, the screening of the cytotoxicity of all the prepared compounds was performed using three cell lines, and the compounds did not show any significant toxic effect.

## 1. Introduction

Pharmaceutics can be administered in different ways depending on their physicochemical properties and pharmacokinetic profile (in general, based on their ADME profile) [1]. The use of skin for the transdermal administration of drugs that can penetrate directly into the system circulation has many advantages, and thus, it is a promising way for administration of many drugs. Unfortunately, transdermal drug delivery frequently encounters the problem of limited penetration of active pharmaceutical ingredients through the skin [2,3]. The skin consists of three basic functional layers: the upper layer (epidermis), curium (dermis), and subcutaneous tissue (hypodermis). The most important and critical for the permeation of all compounds through the skin is the outermost layer of epidermis, the horny layer (stratum corneum, SC), which is, in fact, the real skin barrier [4].

Many different approaches to overcome the skin barrier can be found; from chemical and/or physical modifications of drugs [5] to the specific composition of final drug formulations or application of electrically assisted methods [3,6,7]. The use of chemical permeation enhancers (CPEs), specific pharmaceutical excipients, is one of the possibilities to facilitate drug delivery through the skin. A number of compounds from various chemical classes were investigated as CPEs, many of structurally different compounds expressed transdermal enhancement effect; thus, different sites and mechanisms of actions are supposed [7,8]. In general, all these CPEs are able to modify the SC, and a complex mechanism of action is expected. Many CPEs are particularly small molecules containing characteristic fragments of heteroatoms X−CO−N=, where X is −CH_2_−, −NH_2_, or −NH−, by which they can break intermolecular *H*-bonds among individual components of the SC [7,9,10].

Based on the structural similarity to CPEs [10], (*S*)-8-methyl-6,9-diazaspiro-[4.5]decan-7,10-dione (alaptide, **1**), an original Czech compound prepared by Kasafirek et al. in the 1980s [11,12], was investigated as a CPE with very good results [13,14,15,16,17,18]. Unfortunately, alaptide is very poorly soluble [12,19] and therefore selected “non-spirocyclic” derivatives of alaptide were designed, synthesized, and their transdermal enhancement effects as potential CPEs on the permeation of the model drug theophylline through the skin were investigated.

## 2. Results and Discussion

### 2.1. Chemistry and Physicochemical Properties

**Scheme 1:**
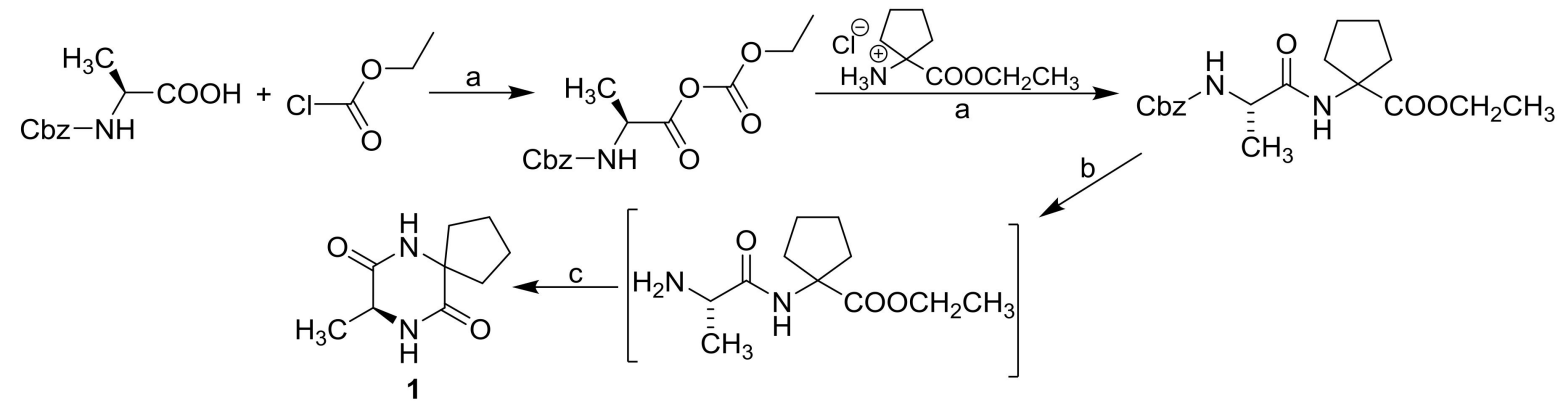
Synthesis of (*S*)-alaptide (**1**). Reagents and conditions: a, 1-ethylpiperidine, dichloromethane; b, H_2_, Pd/C, methanol; c, toluene, acetic acid [20].

Alaptide, originally developed by Kasafirek, was prepared according to Scheme 1 from protected (*S*)-alanine and the ethyl ester of cycloleucine [20].

**Scheme 2:**
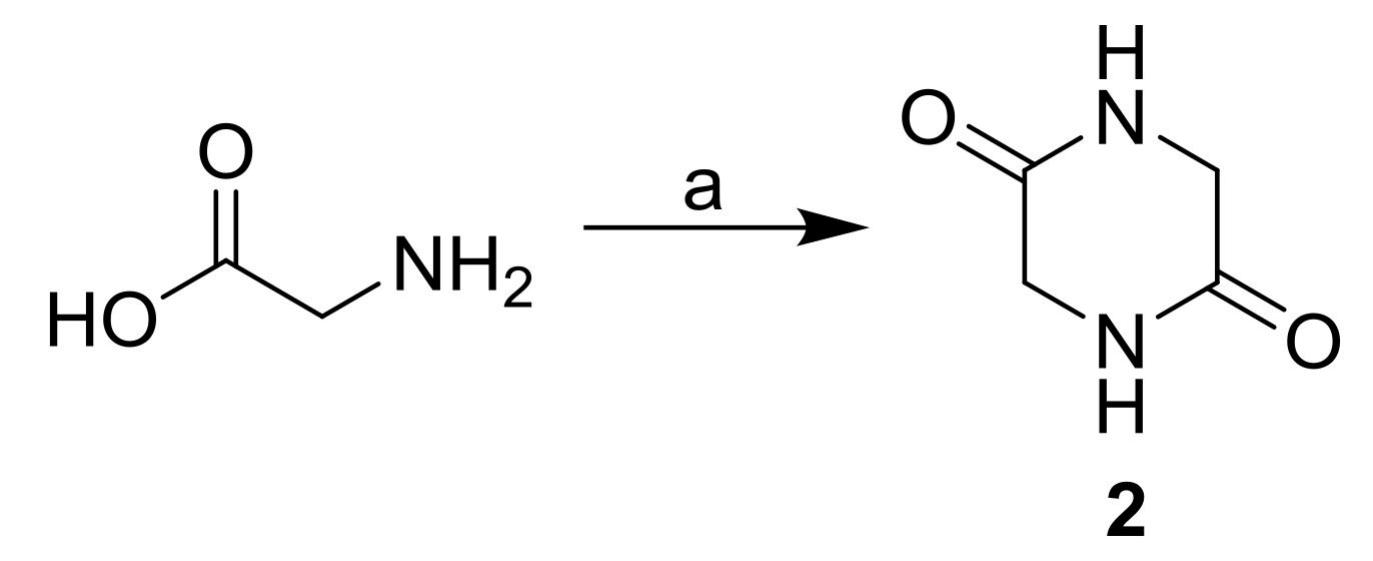
Piperazine-2,5-dione (**2**) synthesis. Reagents and conditions: a, ethylene glycol, 170 °C [21].

**Scheme 3:**
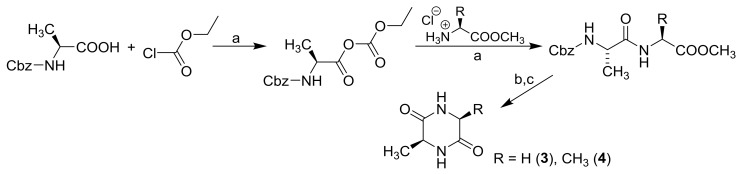
Synthesis of (3*S*)-3-methylpiperazine-2,5-dione (**3**) and (3*S*,6*S*)-3,6-dimethylpiperazine- 2,5-dione (**4**). Reagents and conditions: a, 1-ethylpiperidine, dichloromethane; b, H_2_, Pd/C, methanol; c, toluene, acetic acid.

**Scheme 4:**
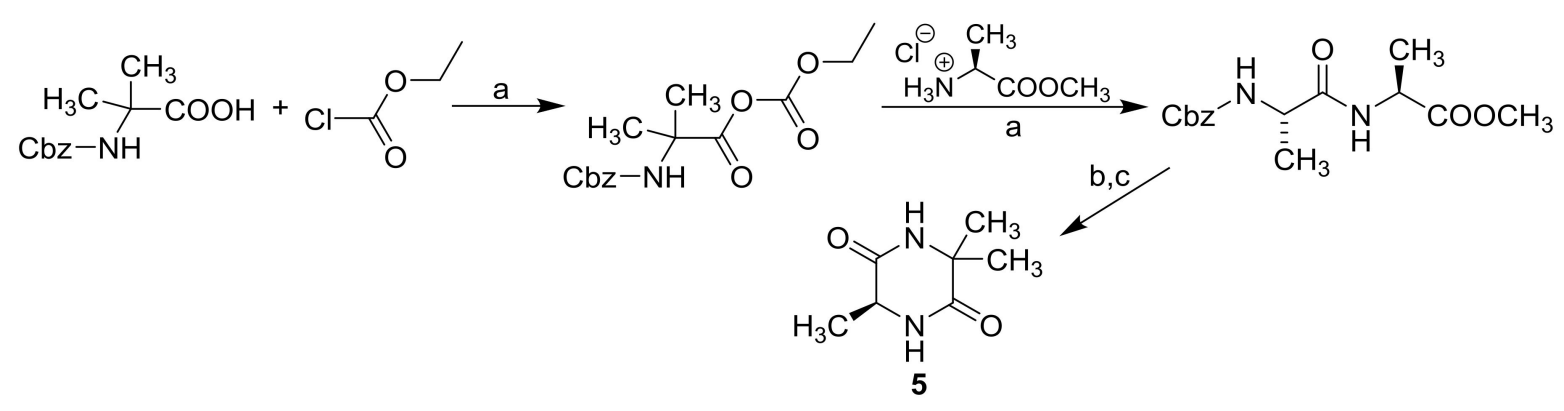
Synthesis of (6*S*)-3,3,6-trimethylpiperazine-2,5-dione (**5**). Reagents and conditions: a, 1-ethylpiperidine, dichloromethane; b, H_2_, Pd/C, methanol; c, toluene, acetic acid.

The syntheses of non-spirocyclic derivatives **2**–**5** are illustrated in Scheme 2, Scheme 3 and Scheme 4. A thermal cyclocondensation of glycine provided piperazine-2,5-dione (**2**) according to the literature procedure [21], see Scheme 2.

The cyclocondensation of the (*S*)-alanine derivative with glycine ester gave (3*S*)-3-methyl- piperazine-2,5-dione (**3**), while with (*S*)-alanine ester, (3*S*,6*S*)-3,6-dimethylpiperazine-2,5-dione (**4**) was obtained. The condensation of 2-methylalanine derivative with (*S*)-alanine ester yielded (6*S*)-3,3,6-trimethylpiperazine-2,5-dione (**5**). For the activation of the benzyloxycarbonyl moiety, the protected amino acids method using the in situ formation of a mixed anhydride with ethyl chloroformate and *N*-ethylpiperidine was applied, similar to the synthesis of alaptide [22]. The attempts to avoid the use of ethyl chloroformate by replacement with benign propylphosphonic anhydride (T3P^®^) were unsuccessful [23,24]. Generated mixed anhydrides were directly used without isolation for a coupling reaction with hydrochlorides of methyl glycinate or methyl l-alaninate to give benzyloxycarbonyl protected dipeptides with an ester at *C*-terminus. The dipeptide-coupled products at deprotection via hydrogenolysis on Pd/C at 3 atm in Parr reactor gave amino dipeptide esters, which then were cyclized in boiling toluene in the presence of acetic acid to form desired molecules **3**–**5**, see Scheme 3 and Scheme 4.

For the synthesis of (3*S*)-3-methylpiperazine-2,5-dione (**3**), [(benzyloxy)carbonyl]-l-alanine and hydrochloride of methyl glycinate were used as starting materials. In case of (3*S*,6*S*)-3,6-dimethyl-piperazine-2,5-dione (**4**), methyl glycinate was replaced by methyl l-alaninate. Finally, (6*S*)-3,3,6-trimethyl-piperazine-2,5-dione (**5**) was prepared from 2-{[(benzyloxy)carbonyl]- amino}-2-methylpropanoic acid and hydrochloride of methyl l-alaninate.

Physicochemical descriptors, such as lipophilicity, solubility, polar surface area, molar volume, parachor, etc., are often used to analyze structure-activity relationships within series of compounds [10,25,26,27,28,29,30,31]; therefore, in many studies dealing with the modification of skin permeation, the effect of these descriptors on permeation was investigated as well (e.g., [7,10] and references therein). Thus, selected characteristics/descriptors were calculated using ACD/Percepta ver. 2012 (Advanced Chemistry Development, Toronto, ON, Canada) and are mentioned in Table 1. In this work, the effect of the following parameters is especially discussed: lipophilicity (expressed as log *P*) and solubility of CPEs in water (expressed as log *S*_W_ at pH 7.4). The accuracy of calculations for simple structures by the ACD/Percepta software used is usually better than 0.2–0.5 logarithmic units. Solubility is not derived primarily from log *P* and not only takes into account pH (solubility as a function of pH) but also compares fragmentary estimates [32] with experimental material from databases of about 6000 compounds. However, it is important to note that the algorithms used for the calculation of the descriptors, in particular, log *P* and log *S*, basically do not take into account configuration and conformation aspects. The positions of substituents are implemented in the calculation protocol; however, the prediction depends on the number of similar compounds in the database. With respect to the importance of permeability and solubility (polarity) for biological activity [33], it is necessary to understand all log *P* and log *S* values predicted by various programs as approximate. In addition, surface tension (ST [dyne/cm]), parachor [cm^3^], and molar volumes (MV [cm^3^]) were calculated as other characteristics of potential CPEs important for affecting the skin.

**Figure 1:**
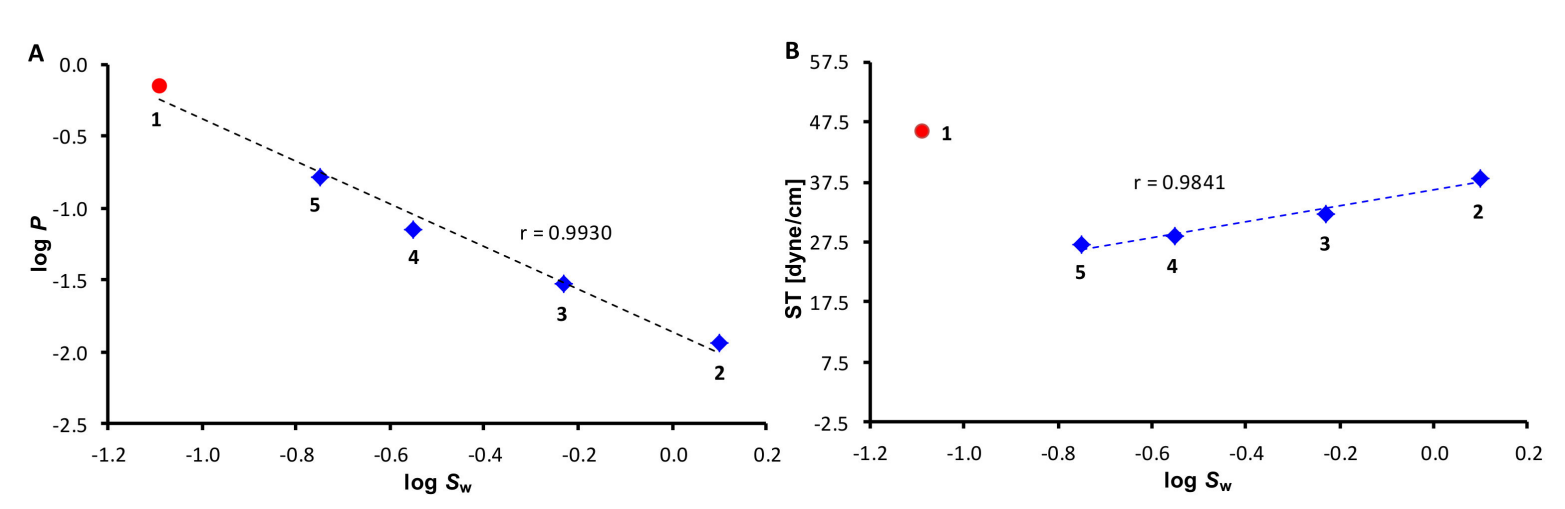
Dependences of lipophilicity (log *P*) (**A**) and surface tension (ST) (**B**) on water solubility (log *S*_w_).

As mentioned above, the solubility of individually tested compounds is important. The dependence of lipophilicity on solubility is illustrated in Figure 1A. The second “spiro” cycle has great influence on lipophilicity as well as solubility, as it is shown by the large distance of compound **1** from the rest of investigated compounds **2**–**5**. Logically, lipophilicity increases (and solubility decreases) with an increasing number of methyl moieties/substituents on the piperazine-2,5-dione scaffold, see Figure 1A. On the other hand, physicochemical and structural similarity of the investigated compounds is evidenced by the high correlation coefficient of the linear dependence log *P* vs. log *S*_w_ (*r* = 0.9930, *n* = 5, the equation of the dependence: log *P* = −1.4846 log *S*_w_ − 1.8602). Similar dependences were also provided by relationships between molar volume or parachor and solubility; therefore, they are not illustrated. Figure 1B shows the dependence of surface tension (ST [dyne/cm]) on solubility (log *S*_w_). Surface tension is inversely related to the surface activity of compounds, i.e., the ability of compounds to act as a detergent (surfactant). The greater the surface tension, the lower the surface activity (the ability of the compound to reduce the surface tension) and the lower the ability of the compound to act as a surfactant. The linear dependence of ST on log *S*_w_ of non-spirocyclic derivatives **2**–**5** shown in Figure 1B can be expressed by the following equation: ST = 13.114 log *S*_w_ + 36.091, *r* = 0.9841, *n* = 4. An increase of surface tension (a decreasing number of methyl substituents on the piperazine-2,5-dione scaffold) increases solubility (log *S*_w_). These discussed parameters play an important role in structure-activity relationships of the investigated piperazine-2,5-diones, as discussed below.

### 2.2. Permeation Experiments

**Figure 2:**
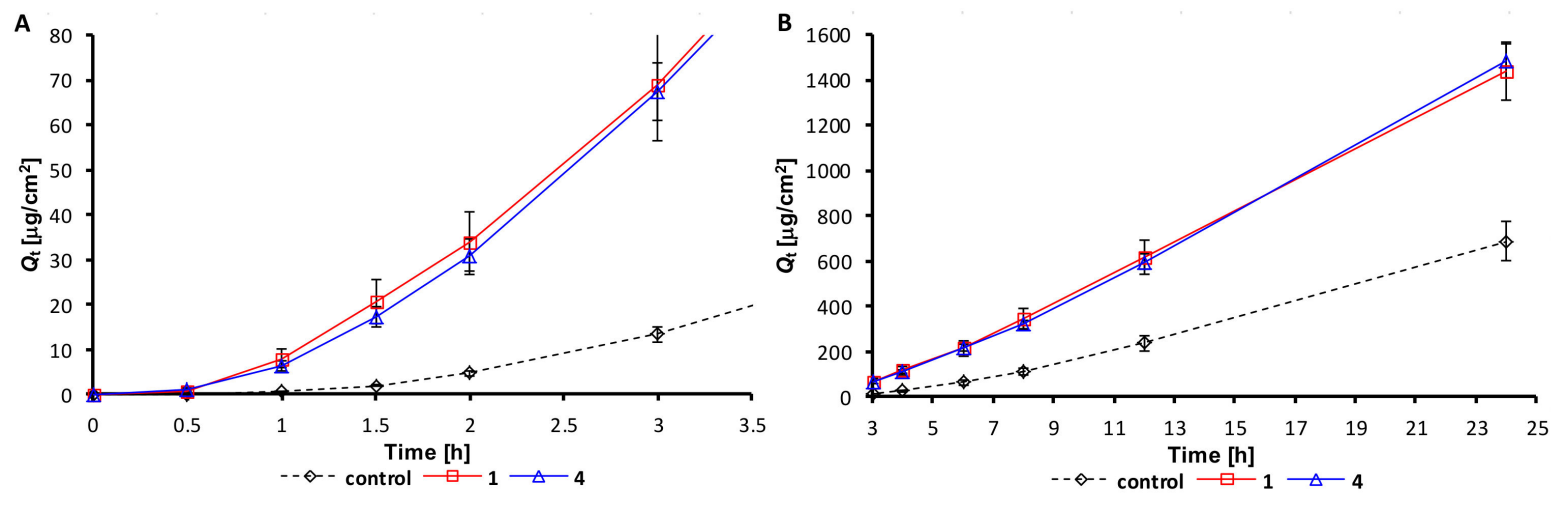
In vitro profile of cumulative permeated amounts *Q*_t_ per unit area [μg/cm^2^] of theophylline alone (control) and after addition of alaptide (**1**) and other tested non-spirocyclic compounds **2**–**5** in ratio 10:1 from propylene glycol/water 1:1 system through the skin in time. *Q*_t_ values are expressed as mean ± SD (*n* = 5 experiments).

The permeation of theophylline through the skin without and with alaptide (**1**) as a model molecule and with other prepared non-spirocyclic derivatives of alaptide **2**–**5** was tested from the propylene glycol/water (1:1) system as a donor vehicle using static Franz diffusion cells [34] within 24 h. It should be noted that recently reported studies demonstrated that propylene glycol or a propylene glycol/water system does not interfere with membranes [35,36]. Theophylline was used as a model penetrant/drug of medium polarity (log *P* = −0.06) [37,38], as it has been comprehensively investigated in transdermal penetration experiments [39,40,41]. Full-thickness pig ear skin was chosen for the In vitro evaluation of permeation. This tissue is considered as a suitable in vitro model of the human skin [42,43,44], as pig skin is histologically and biochemically similar to the human skin [45]. The values obtained from the permeation experiments were expressed as the cumulative permeated amount of the drug (*Q*_t_ [μg]) per unit of skin surface area, see Table 2. The dependences of the cumulative permeated amount of the drug per unit of skin surface area in time for the control and the most effective compounds **1** and **4** are illustrated in Figure 2, which is divided into parts A and B for better lucidity.

It is evident that the addition of piperazine-2,5-diones **1**–**5** caused the enhancement of theophylline permeation. Based on the above-mentioned results, the permeated amount of theophylline with all the tested CPEs increased rapidly already at minute 30. The permeated amount of theophylline with the CPEs at minute 60 reached approx. 5- to 15-fold higher values than from the formulation without the CPEs, see Table 2, and compounds **1** and **4** were the most effective. Similarly, a sharp enhancement of transdermal permeation was found at permeation of other drugs from different classes [13,14,15,16,17,18]. On the other hand, the enhancement effect (the slope of permeation) of all the tested CPEs began to decrease from the 4th hour, see Figure 2B. Nevertheless, it can be stated that theophylline without the CPEs permeated moderately in comparison with theophylline with added CPEs. Within the whole investigated time range, the corresponding *Q*_t_ values of samples with CPEs **1**–**5** were considerably higher in every time than those estimated in the system without CPEs (control sample), see Table 2. The *Q*_t_ values related to propylene glycol:water systems with alaptide (**1**) and compound **4** were within experimental error, see Figure 2, and the mean values of the permeated amount of theophylline with derivative **3** demonstrated that the enhancement activity of compound **3** was the lowest.

The effectivity of alaptide (**1**) and (3*S*,6*S*)-3,6-dimethylpiperazine-2,5-dione (**4**) as potential CPEs can also be confirmed by other calculated permeation parameters of theophylline without and with enhancers from propylene glycol:water (1:1), such as steady-state permeation fluxes (*J* [μg/h/cm^2^]), lag times (*t*_lag_ [h]), permeability coefficients (*K*_p_ [cm/h]), and enhancement ratios (*ER*) that are mentioned in Table 3 (for the time period 1–3 h, pseudo-steady state values marked by an apostrophe) and in Table 4 (for the time period 6–12 h, real steady state).

Parameters calculated for the time period 1–3 h mentioned in Table 3 are more important than the standard steady-state parameters calculated for the time period 6–12 h (Table 4), because they reflect a significantly increased observed enhancement ratio (*ERs’* ca. 4.4) immediately after application (*ERs* compared with *ERs’* are approx. half: 51.3% (**1**), 51.4% (**2**), 56.2% (**3**), 50.6% (**4**), 67.5% (**5**)), which is more favorable than long-term application due to the stability of semi-solid drug dosage forms and compliance of patients. These facts indicate that not only the pattern alaptide (**1**) but also dimethylpiperazine-2,5-dione derivative **4** is able to enhance the permeation of theophylline through the skin.

It can be assumed that the enhancement activity of individually tested compounds is dependent on their physicochemical properties, as mentioned above. Within non-spirocyclic derivatives **2**–**5**, it is evident that the enhancement effect is negatively influenced by asymmetric substitution of the basic scaffold, i.e., 3-methylpiperazine-2,5-dione (**3**) showed the lowest activity followed by 3,3,6-trimethylpiperazine-2,5-dione (**5**). It seems that the position and number of methyl moieties is crucial for the activity. Besides these structural parameters, the activity of effective compounds is partially affected by lipophilicity and by molar volume; activity increases with increasing log *P* and bulkiness and is inversely influenced by solubility, see Table 1 and Table 2. Thus, it can be stated that the same as for all drugs, also for these types of excipients, lipo-hydrophilic properties must be balanced. On the other hand, alaptide (**1**) has significantly higher surface tension value than comparable effective derivative **4**. Unsubstituted derivative **2** with medium activity is between them. It can be only hypothesized if these “opposite” values of compound **1** and **4** have strong impact on their enhancement activity, which may result in a different mode of action. Similarly, it can be speculated about the enhancement effect of piperazine-2,5-diones substituted by a longer alkyl tail(s), as, for example, is discussed for similar compounds in [10]. On the other hand, toxicity, skin irritability, and hydrophobicity increase, while solubility decreases with increasing surface activity [7].

### 2.3. In Vitro Cytotoxicity Assay

The cytotoxicity of the tested compounds was evaluated using a normal human skin fibroblast cells (BJ), a T-lymphoblastic leukaemia cell line (CEM), and a breast adenocarcinoma cell line (MCF7). The cytotoxicity was expressed as the IC_50_ value (compound concentration causing 50% inhibition of cell population proliferation). A compound is considered as cytotoxic when it demonstrates a toxic effect on cells at the concentration up to 10 μM [46], and the highest tested concentration that was used for the toxicity assay was five times this value. Treatment with 50 μM of the discussed compounds did not express any cytotoxic effect on any of the tested cell lines. Based on the results of these observations, it can be concluded that the discussed alaptide (**1**) as well as other piperazine-2,5-diones **2**–**5** can be considered as non-toxic agents.

## 3. Materials and Methods

### 3.1. General Information

All reagents were purchased from Merck (Sigma-Aldrich, St. Louis, MO, USA) and Acros Organics (Thermo Fisher Scientific, Geel, Belgium). The melting points were determined on a Kofler hot-plate apparatus HMK (Franz Kustner Nacht KG, Dresden, Germany) and are uncorrected. The measurement of optical rotations was carried on Automatic polarimeter AA-10 (Optical Activity, Ramsey, UK). The concentration of samples is given in 10 mg/mL. Infrared (IR) spectra were recorded on a Smart MIRacle™ ATR ZnSe for Nicolet™ Impact 410 Fourier-transform IR spectrometer (Thermo Scientific, West Palm Beach, FL, USA). The spectra were obtained by the accumulation of 256 scans with 2 cm^−1^ resolution in the region of 4000–650 cm^−1^. All ^1^H- and ^13^C-NMR spectra were recorded on a JEOL ECZR 400 MHz NMR spectrometer (400 MHz for ^1^H and 101 MHz for ^13^C, Jeol, Tokyo, Japan) in dimethyl sulfoxide-*d*_6_ (DMSO-*d*_6_). ^1^H and ^13^C chemical shifts (δ) are reported in ppm. The signal of the residual solvent (DMSO-*d*_6_) was used as a reference. High-resolution mass spectra were measured using a high-performance liquid chromatograph Dionex UltiMate^®^ 3000 (Thermo Scientific) coupled with an LTQ Orbitrap XL^TM^ Hybrid Ion Trap-Orbitrap Fourier Transform Mass Spectrometer (Thermo Scientific) equipped with a HESI II (heated electrospray ionization) source in the positive mode.

### 3.2. Synthesis

Alaptide, (*S*)-8-methyl-6,9-diazaspiro[4.5]decan-7,10-dione, (**1**) was prepared according to Kasafirek et al. [20].

*Piperazine-2,5-dione* (**2**) [21]. Glycine (7.5 g, 0.1 mol) was suspended in 50 mL of anhydrous ethylene glycol, and the mixture was stirred and heated at 170–180 °C for 6 h. After cooling and standing in a refrigerator overnight, the precipitate was filtered off and washed with methanol. Crude product was purified by recrystallization from hot water and dried under reduced pressure. Yield 62%; Mp >280 °C; IR (cm^−1^): 3047, 2914, 2873, 1673, 1468, 1441, 1338, 1073, 914, 835, 806, 669; ^1^H-NMR (DMSO-*d*_6_), δ: 7.95 (br. s., 2H), 3.71 (d, *J* = 2.1 Hz, 4H); ^13^C-NMR (DMSO-*d*_6_), δ: 166.1, 44.3; HR-MS: for C_4_H_7_N_2_O_2_ [M + H]^+^ calculated 115.0502 *m*/*z*, found 115.0495 *m*/*z*.

General procedure for preparation of piperazine-2,5-diones **3**–**5**. In a 3-neck round bottom flask equipped with a septum, a thermometer and an adapter for a balloon with argon, Cbz-aminoacid (1.0 equiv., 5.0 mM) and *N*-ethylpiperidine (1.1 equiv., 5.5 mM) were dissolved in anhydrous methylene chloride (25 mL) and cooled to −10 °C. Ethyl chloroformate (1.1 equiv., 5.5 mM) was added at the same temperature, and the mixture protected from ingress of moisture was stirred for 2 h. Hydrochloride of methyl l-alaninate or methyl glycinate (1.0 equiv., 5.0 mM) was dissolved in the second flask in anhydrous methylene chloride (10 mL) and cooled to 0 °C, and then *N*-ethylpiperidine (1.0 equiv., 5.0 mM) was added. The whole volume of the second flask was immediately transferred to a 3-neck round bottom flask with the help of syringe, and additional *N*-ethylpiperidine (1.0 equiv., 5.0 mM) was added. The solution was stirred at the same temperature for 2 h and then left at 5 °C overnight. After that, the reaction mixture was quenched with water (35 mL). The aqueous phase was extracted with methylene chloride (3 × 15 mL). Combined organic phases were washed with 2M HCl solution (3 × 10 mL), water (10 mL), saturated sodium hydrogen carbonate solution (3 × 10 mL), and brine (10 mL) and dried over anhydrous sodium sulfate. After filtration, the solvent was removed under reduced pressure to give Cbz-amino dipeptide esters. The crude Cbz-amino dipeptide esters were hydrogenated with 60 mL of methanol and 10% Pd/C as a catalyst (0.5 g of Pd/C per 3 mM of intermediate dipeptide esters) at room temperature. The reactor (250 mL capacity Parr reactor) was pressurized with hydrogen to 3 bars at ambient temperature. Reactor pressure remained unchanged during the reaction period of 1 h. After releasing the pressure, the catalyst was removed by filtration through Celite, and the solvent was removed under reduced pressure. The crude amino dipeptide esters were placed into a 250 mL round-bottomed flask equipped with an egg-shaped, Teflon-coated magnetic stirring bar and a reflux condenser, and toluene (100 mL) containing 5 mL of acetic acid was added. The reaction mixture was stirred and refluxed for 2 h, then the reflux condenser was removed, and the amount of the solvent was reduced to 10% at atmospheric pressure. The remaining solvent was removed under reduced pressure and the residue was recrystallized from propane-2-ol. Pure piperazine-2,5-diones **3**–**5** were dried at 40 °C under reduced pressure.

*(3S)-3-Methylpiperazine-2,5-dione* (**3**) [47] was prepared according to the general procedure described above from [(benzyloxy)carbonyl]-l-alanine and hydrochloride of methyl glycinate. Yield 54%; Mp 278–280 °C; [α]^25^_D_: −4.0 (CH_3_OH), −5 (H_2_O) [48] or −3.78 (H_2_O) [49]; IR (cm^−1^): 3047, 2879, 1663, 1456, 1327, 1094, 1044, 802, 669; ^1^H-NMR (DMSO-*d*_6_), δ: 8.09 (br. s., 1H), 7.90 (br. s., 1H), 3.84 (dq, *J* = 0.9, 7.0 Hz, 1H), 3.78–3.67 (m, 2H), 1.26 (d, *J* = 7.0 Hz, 3H); ^13^C-NMR (DMSO-*d*_6_), δ: 168.8, 166.2, 49.7, 44.5, 18.6; HR-MS: for C_5_H_9_N_2_O_2_ [M + H]^+^ calculated 129.0659 *m*/*z*, found 129.0652 *m*/*z*.

*(3S,6S)-3,6-Dimethylpiperazine-2,5-dione* (**4**) [50] was prepared according to the general procedure described above from [(benzyloxy)carbonyl]-l-alanine and hydrochloride of methyl l-alaninate. Yield 58%; Mp 282–284 °C; [α]^25^_D_: −20.0 (CH_3_OH), −24 (H_2_O)[48]; IR (cm^−1^): 3084, 2987, 1665, 1450, 1375, 1307, 1091, 945, 831, 672; ^1^H-NMR (DMSO-*d*_6_), δ: 8.05 (br. s., 1H), 3.91 (dq, *J* = 1.0, 7.0 Hz, 1H), 1.26 (d, *J* = 7.0 Hz, 3H); ^13^C-NMR (DMSO-*d*_6_), δ: 168.0, 49.8, 18.8; HR-MS: for C_6_H_11_N_2_O_2_ [M + H]^+^ calculated 143.0815 *m*/*z*, found 143.0803 *m*/*z*.

*(6S)-3,3,6-Trimethylpiperazine-2,5-dione* (**5**) [51] was prepared according to the general procedure described above from 2-{[(benzyloxy)carbonyl]amino}-2-methylpropanoic acid and hydrochloride of methyl l-alaninate. Yield 49%; Mp 271–273 °C; [α]^25^_D_: +1.0 (CH_3_OH); IR (cm^−1^): 3181, 3043, 1666, 1438, 1381, 1311, 1241, 1199, 1164, 1098, 932, 835, 771, 683 ^1^H-NMR (DMSO-*d*_6_), δ: 8.10 (br. s., 1H), 7.97 (br. s., 1H), 3.93 (dq, *J* = 1.2, 6.9 Hz, 1H), 1.30 (s, 6H), 1.27 (d, *J* = 7.0 Hz, 3H); ^13^C-NMR (DMSO-*d*_6_), δ: 170.7, 168.1, 55.0, 49.9, 27.4, 27.4, 19.0; HR-MS: for C_7_H_13_N_2_O_2_ [M + H]^+^ calculated 157.0972 *m*/*z*, found 157.0964 *m*/*z*.

### 3.3. In Vitro Transdermal Permeation Experiments

Skin samples were obtained from porcine ear. Full-thickness pig (*Sus scrofa* f. *domestica*) ear skin was cut in fragments and stored at −20 °C until utilized. Skin samples were slowly thawed (at 4 °C overnight and then at ambient temperature) before each experiment [52,53]. The penetration enhancing effect of alaptide was evaluated In vitro, using a vertical Franz diffusion cell (SES-Analytical Systems, Bechenheim, Germany) with a donor surface area of 0.6359 cm^2^ and a receptor volume of 5.2 mL, see Figure 1. The skin was mounted between the donor and receptor compartments of the Franz diffusion cell with the epidermal side up. The receptor compartment was filled with phosphate buffered saline (pH 7.4) and maintained at 34.0 ± 0.5 °C [34,52] using a circulating water bath. The receptor compartment content was continuously stirred using a magnetic stirring bar. The skin was kept in contact with the receptor phase for 0.5 h prior to the experiment. Theophylline was purchased from Sigma-Aldrich; all other reagents and solvents were purchased from Merck (Darmstadt, Germany). Donor samples were prepared by dissolving theophylline (10 mg) and the tested CPE (1 mg) in propylene glycol (0.5 mL), to which water (0.5 mL) was added consequently to create a mixture of propylene glycol/water 1:1. These mixtures were shaken vigorously and then sonicated for 10 min at 40 °C; then these stable systems (dissolved theophylline in the enhancer emulsion) were applied to the skin surface, and the donor compartment of the cell was covered by Parafilm^®^. Control samples were prepared in the same manner without the tested CPE. Samples (0.5 mL) of the receptor phase were withdrawn at pre-determined time intervals (30, 60, 90, 120, 180, 240, 360, 480, 720 and 1440 min), and the cell was refilled with an equivalent amount of fresh buffer solution. A minimum of five determinations were performed using skin fragments from at least 2 animals for each compound, and the data was expressed as means ± SD.

The analysis of the samples was performed using an Agilent 1200 series HPLC system, equipped with a diode array detection system, a quaternary model pump, and an automatic injector (Agilent Technologies, Santa Clara, CA, USA). Data acquisition was performed using ChemStation chromatography software (B.04.01 LC 1200, Agilent Technologies). A Gemini C6-Phenyl 110A 5 μm, 250 × 4.6 mm (Phenomenex, Torrance, CA, USA) chromatographic column was used. The total flow of the column was 1.0 mL/min; injection was 10 μL; column temperature was 40 °C; and sample temperature was 20 °C. The detection wavelength of 272 nm was chosen; the time of analysis was 7 min. A mixture of acetonitrile (HPLC grade, 10.0%), methanol (HPLC grade, 15.0%), and H_2_O (HPLC—Mili-Q Grade, 75.0%) was used as the mobile phase. The retention time (t_R_) of theophylline was 4.36 ± 0.05 min; the limit of detection (LOD) was 0.0063 µg/mL; and the limit of quantification (LOQ) was 0.0209 µg/mL. An equation of calibration curve was y = 30.59585x, and the correlation coefficient was *r* = 0.9998 (*n* = 7).

As a result of the sampling, the receptor compartment concentration of the tested CPEs was corrected for sample removal and replenishment using equation: *C*′_n_ = *C*_n_ (*V*_t_/*V*_t_−*V*_s_) (*C*′_n−1_/*C*_n−1_), where *C*′_n_ = corrected drug concentration in the n^th^ sample, *C*_n_ = measured drug concentration in the n^th^ sample, *C*′_n-1_ = corrected drug concentration in the (n−1)^th^ sample, *C*_n−1_ = measured drug concentration in the (n−1)^th^ sample, *V*_t_ = total volume of receptor solution, *V*_s_ = volume of the sample, and *C*′_1_ = *C*_1_. The corrected data were expressed as the cumulative drug permeation (*Q*_t_) per unit of skin surface area using the equation: *Q*_t_ = *C*′_n_/*A*, where *A* = 0.6359 cm^2^ in the experiment. From the slope of the linear portion of the curve representing the dependence of the cumulative amount of the drug (*Q*_t_ [μg]) per unit area on time (*t* [h]), steady-state permeation flux (*J* [μg/h/cm^2^]) was determined. Similarly, the lag time (*t*_lag_ [h]) was determined by extrapolating the linear portion of the cumulative amount of permeation per unit area (*Q*_t_) versus time (*t* [h]) curve to the abscissa. The permeability coefficient (*K*_p_ [cm/h]) can be calculated according to *K*_p_ = *J*/*C*_d_, where *C*_d_ = drug concentration in the donor compartment. It is assumed that under sink conditions, drug concentration in the receptor compartment is negligible compared to that in the donor compartment. The enhancement effect was expressed as an enhancement ratio (*ER*) that was calculated by the formula *ER* = *J*_ss−x_/*J*_ss−k_, where *J*_ss−x_ = steady-state permeation flux with CPE, *J*_ss−k_ = steady-state permeation flux without CPE [1,54,55]. All the calculated data are listed in Table 1, Table 2 and Table 3 and illustrated in Figure 2.

### 3.4. In Vitro Cytotoxicity Assay

Dulbecco’s modified Eagle’s medium (DMEM), l-glutamine, trypsin, penicillin, and streptomycin were purchased from Sigma (St. Louis, MO, USA); fetal bovine serum (FBS) and Calcein AM were purchased from Invitrogen (Carlsbad, CA, USA). The cell lines used for screening, T-lymphoblastic leukemia CEM, breast adenocarcinoma MCF7, and human foreskin fibroblasts BJ, were obtained from the American Type Culture Collection (Manassas, VA, USA). Cells were cultured in DMEM medium (Sigma). All media used were supplemented with 10% heat-inactivated fetal bovine serum, 2 mM l-glutamine, 10,000 U penicillin, and 10 mg/ml streptomycin. The cell lines were maintained under standard cell culture conditions at 37 °C and 5% CO_2_ in a humid environment. Cells were subcultured two or three times a week using the standard trypsinization procedure. Suspensions with approximately 1.0 × 10^5^ cells/mL (0.5 × 10^5^ cells/mL for BJ) were distributed in 96-well microtiter plates, and after 12 h of stabilization, the tested compounds were added at desired concentrations in Lutrol F 127. Control cultures were treated with Lutrol F 127 alone, and the final concentration of Lutrol F 127 in the reaction mixture never exceeded 0.5%. In most cases, six serial 3-fold dilutions of the test substances were added at time zero in 20 µL aliquots to the microtiter plate wells, and the highest final concentration in the wells was 50 µM. After incubation for 72 h, Calcein AM solution (2 µM, Molecular Probes, Invitrogen, CA, USA) was added, and the cells were incubated for a further hour. The fluorescence from viable cells was then quantified, using a Fluoroskan Ascent fluorometer (Labsystems, Vantaa, Finland). The percentage of surviving cells in each well was calculated by dividing the intensity of the fluorescence signals from the exposed wells by the intensity of signals from control wells and multiplying by 100. These ratios were then used to construct dose-response curves from which IC_50_ values were calculated.

## 4. Conclusions

A series of four selected piperazine-2,5-diones as non-spirocyclic analogues of the pattern compound named alaptide (**1**, (*S*)-8-methyl-6,9-diazaspiro[4.5]decan-7,10-dione) were synthesized and characterized. The ability of all the compounds to enhance the permeation of the model drug theophylline from the medium of propylene glycol:water (1:1) was investigated using vertical Franz diffusion cells and a full-thickness pig (*Sus scrofa* f. *domestica*) ear skin. All the compounds were tested on their cytotoxic effect using human foreskin fibroblasts and two cancer cell lines, and none of the compounds expressed any toxic effect. All the compounds enhanced the permeation of theophylline through the skin, e.g., the permeated amount of theophylline was already at the 1st h after application approx. 1500% and 1200% higher at the application of alaptide (**1**) and (3*S*,6*S*)-3,6-dimethylpiperazine-2,5-dione (**4**), respectively, than from the formulation without the tested compounds. The position and number of methyl moieties as well as balanced lipo-hydrophilic properties seem to be crucial for the activity. The contributions of compounds **1** and **4** to the enhanced permeation of theophylline through the skin is significant not only in case of long-term application but especially immediately after application, which is favorable in terms of the stability of semi-solid drug dosage forms and compliance of patients. The exact mechanisms of actions of these piperazine-2,5-diones have not been known but have been under investigation.

## Figures and Tables

**Table 1:** Structure and calculated (ACD/Percepta ver. 2012) physicochemical properties of alaptide (**1**) and its derivatives **2**–**5**: lipophilicity (log *P*), water solubility (log *S*_w_), molar volume (MV [cm^3^]), parachor [cm^3^], and surface tension (ST [dyne/cm]).

Comp.	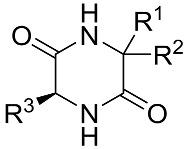							
	R^1^	R^2^	R^3^	log *P*	log *S*_w_	MV [cm^3^]	Parachor [cm^3^]	ST [dyne/cm]
**1**	–(CH_2_)_4_–		−CH_3_	−0.15	−1.09	156.64	392.52	45.88
**2**	−H	−H	−H	−1.94	0.10	91.5	227.40	38.12
**3**	−H	−H	−CH_3_	−1.53	−0.23	109.04	265.43	32.10
**4**	−CH_3_	−H	−CH_3_	−1.15	−0.55	126.22	303.45	28.36
**5**	−CH_3_	−CH_3_	−CH_3_	−0.79	−0.75	142.53	341.84	27.03

**Table 2:** Cumulative permeated amounts *Q*_t_ of theophylline per unit area [μg/cm^2^] from propylene glycol:water 1:1 without (control) and with CPEs **1–5** achieved in In vitro transdermal permeation experiments using Franz diffusion cell. *Q*_t_ values are expressed as mean ± SD (*n* = 5 experiments).

Time [h]	Control	Compounds				
		1 (alaptide)	2	3	4	5
**0.5**	0.00 ± 0.00	0.79 ± 0.58	0.40 ± 0.24	0.39 ± 0.13	0.93 ± 0.18	0.32 ± 0.17
**1**	0.53 ± 0.12	7.92 ± 2.18	4.70 ± 1.17	2.52 ± 1.37	6.29 ± 1.01	2.44 ± 1.69
**1.5**	1.89 ± 0.33	20.53 ± 4.88	12.92 ± 2.08	7.54 ± 3.43	17.29 ± 2.37	8.22 ± 4.42
**2**	4.62 ± 0.60	33.66 ± 7.04	23.76 ± 3.04	14.77 ± 6.20	30.83 ± 3.59	16.16 ± 7.21
**3**	13.36 ± 1.63	68.73 ± 12.20	52.62 ± 5.18	34.41 ± 11.47	67.37 ± 6.53	39.36 ± 14.07
**4**	26.07 ± 3.31	114.08 ± 17.92	86.13 ± 7.16	59.64 ± 17.67	110.57 ± 9.03	69.44 ± 21.40
**6**	62.00 ± 8.40	212.49 ± 3062	168.24 ± 11.85	125.63 ± 28.83	216.87 ± 14.89	151.18 ± 37.41
**8**	112.06 ± 14.78	341.30 ± 45.25	270.85 ± 16.94	191.59 ± 37.56	319.76 ± 17.95	234.74 ± 49.44
**12**	235.20 ± 34.88	616.34 ± 72.86	496.43 ± 32.00	368.53 ± 56.04	595.79 ± 31.81	460.21 ± 77.54
**24**	684.56 ± 87.05	1439.30 ± 127.94	1253.20 ± 108.70	998.62 ± 120.08	1482.83 ± 74.42	1273.10 ± 144.46

**Table 3:** Pseudo-permeation parameters of theophylline in range 1–3 h without (control) and with tested CPEs **1**–**5** from propylene glycol:water (1:1): steady-state permeation fluxes (*J’*), corresponding lag times (*t*_lag_*’*), permeability coefficients (*K*_p_′), and enhancement ratios (*ERs’*). All values are expressed as mean ± SD (*n* = 5 experiments).

Comp.	*J’_1-3h_* [μg/cm^2^/h]	*t*_lag_*’_1-3h_* [h]	*K*_p_*’_1-3h_*×10^-3^ [cm/h]	*ERs’_1-3h_*
control (theophylline)	6.60 ± 0.80	1.10 ± 0.02	0.66 ± 0.08	1.00 ± 0.05
**1**	29.84 ± 7.02	0.77 ± 0.14	2.98 ± 0.70	4.52 ± 1.06
**2**	24.33 ± 2.05	0.91 ± 0.04	2.43 ± 0.20	3.68 ± 0.31
**3**	16.55 ± 3.92	0.87 ± 0.13	1.66 ± 0.39	2.51 ± 0.59
**4**	28.70 ± 1.73	0.80 ± 0.12	2.87 ± 0.17	4.35 ± 0.26
**5**	17.49 ± 5.48	0.91 ± 0.16	1.75 ± 0.55	2.65 ± 0.83

**Table 4:** Permeation parameters of theophylline in range 6–12 h without (control) and with tested CPEs **1**–**5** from propylene glycol:water (1:1): steady-state permeation fluxes (*J*), corresponding lag times (*t*_lag_), permeability coefficients (*K*_p_), and enhancement ratios (*ERs*). All values are expressed as mean ± SD (*n* = 5 experiments).

Comp.	*J_ss_* [μg/cm^2^/h]	*t*_lag_ [h]	*K*_p_ × 10^−3^ [cm/h]	*ERs*
control (theophylline)	29.12 ± 3.93	3.74 ± 0.41	2.91 ± 0.39	1.00 ± 0.05
**1**	67.52 ± 9.19	2.91 ± 0.17	6.75 ± 0.92	2.32 ± 0.32
**2**	54.94 ± 3.46	2.99 ± 0.06	5.49 ± 0.35	1.89 ± 0.12
**3**	41.02 ± 4.77	3.13 ± 0.41	4.10 ± 0.48	1.41 ± 0.16
**4**	63.99 ± 3.25	2.77 ± 0.16	6.40 ± 0.33	2.20 ± 0.11
**5**	52.20 ± 6.74	3.31 ± 0.33	5.22 ± 0.67	1.79 ± 0.23
